# Hospitalized patients' health‐related social needs: A comparison of screenings conducted by hospital staff and research staff

**DOI:** 10.1002/jhm.70164

**Published:** 2025-08-20

**Authors:** Kevin J. O'Leary, Teresa Pollack, Cynthia Barnard, Jane S. Kim, Lauren Leviton, Luke Favia, Tara Lagu, Carol Haywood

**Affiliations:** ^1^ Division of Hospital Medicine Northwestern University Feinberg School of Medicine Chicago Illinois USA; ^2^ Northwestern Memorial Healthcare Chicago Illinois USA; ^3^ Center for Health Services and Outcomes Research Northwestern University Feinberg School of Medicine Chicago Illinois USA; ^4^ Determinants of Health Division, Department of Medical Social Sciences Northwestern University Feinberg School of Medicine Chicago Illinois USA

## Abstract

New policies require hospitals to identify and address patients' health‐related social needs (HRSN) yet provide minimal guidance on how screening should be conducted. This observational study occurred at a large academic hospital serving a diverse population. Hospital and research staff independently screened newly admitted medical patients for six HRSNs using an instrument designed by a quality improvement team. We calculated *κ* statistics to assess HRSN agreement between hospital and research staff. Analysis of 413 patients revealed fair to moderate agreement between hospital and research staff screenings (*κ* = 0.25–0.54). Hospital staff identified fewer patients with needs related to medication affordability, housing, food, transportation, and mental health, but identified a higher proportion with needs related to the usual source of care. Hospital staff underestimate the proportion of patients with HRSN, potentially excluding patients from referral to social services.

## BACKGROUND

Acknowledging the large effect of upstream social factors on downstream health outcomes, national stakeholders have developed policies for healthcare organizations to identify and address health‐related social needs (HRSN). The Joint Commission now requires accredited hospitals to assess patients' HRSNs and provide information about community resources and support services.^1^, ^2^ Similarly, the Centers for Medicare and Medicaid Services (CMS) Inpatient Quality Reporting Program requires hospitals to report performance in screening for food insecurity, housing instability, transportation needs, utility difficulties, and interpersonal safety.^3^ Unfortunately, little is known about the effectiveness of strategies used to screen for social needs. A recent scoping review of the collection of social determinants of health data in inpatient general internal medicine wards found that a variety of screening tools were used among included studies, and limited information was provided describing data collection methods.^4^ Notably, policymakers provide minimal guidance on how screening should be conducted and allow hospitals to implement self‐selected screening instruments by any role(s) of the hospital's choosing and at any point during hospitalization.

The manner in which screening is conducted, and the screening instrument used may influence the effectiveness of HRSN screening in various ways. Our research team has conducted HRSN screening of patients hospitalized with medical conditions since 2019.^5^ When our hospital began HRSN screening in May 2021, we sought to assess HRSN screening effectiveness by comparing (1) hospital staff and research staff screening using the same instrument and (2) research staff screening using two different instruments.

## METHODS

### Study design and setting

This cross‐sectional observational study was conducted on the general medicine services at Northwestern Memorial Hospital, a large urban academic hospital that serves a diverse adult patient population. This study included patients hospitalized from May 2021 through April 2024.

### Research team health‐related social needs screening

A research coordinator conducted structured interviews each weekday on patients' second or third hospital day. Patients with cognitive impairment, limited English proficiency, or other communication barriers were excluded. After obtaining informed consent, the research coordinator interviewed patients using the screening instrument used by hospital staff. The research coordinator also administered the Accountable Health Communities Health‐Related Social Needs (AHC HRSN) questionnaire, a tool developed by CMS.^6^ The interview took approximately 10‐15 min to complete, and participants were not compensated.

### Health system health‐related social needs screening

Newly hospitalized patient were screened for six social needs by their assigned nurse during the admission assessment. The hospital screening instrument was developed by a quality improvement team based on a review of published instruments and identified HRSNs related to usual source of medical care (i.e., doctor or clinic where patient usually goes for medical care), medication affordability, housing instability, food insecurity, transportation needs, and access to mental health services (see Appendix). Nurses documented the presence or absence of each social need in the patient's electronic health record (EHR). If one or more social needs were present, the nurse asked the patient if they wanted assistance and, if so, consulted the unit's social worker. Social workers met with patients to assess social needs and updated the screening data in the EHR.

### Statistical analysis

We merged data collected by research coordinators with EHR data, including HRSN screening data collected during the patient's hospital stay. We did not include HRSN screening data collected before the patient's hospitalization (e.g., in an outpatient encounter or prior hospitalization) unless screening data were reassessed and documented during the hospitalization. We calculated descriptive statistics of patient characteristics. We report the percentage of patients with each social need as recorded by hospital and research staff. We calculated *κ* statistics to assess agreement between hospital staff and research staff on the presence of social needs using the hospital screening instrument. We also calculated *κ* statistics on data collected by research staff for the three social needs assessed by both the hospital screening instrument and the AHC HRSN tool (housing instability, food insecurity, and transportation needs).

## RESULTS

### Patient enrollment and characteristics

Of 4,325 patients assessed for eligibility, 2432 were excluded, and 838 declined to participate (see Appendix Figure). The remaining 1055 patients completed HRSN screens by research staff. Of these, 628 patients were excluded due to missing HRSN screens conducted by hospital staff, and 14 patients were excluded due to missing HRSN elements in screenings conducted by research staff, leaving 413 patients for analysis. Patients were a mean 60.1 ± 16.7 years of age, majority white race (250/413; 60.5%), and majority insured by Medicare (223/413; 54.0%) (see Appendix Table 1).

### Agreement based on screening approach and instrument used

Analysis revealed fair to moderate agreement between hospital and research staff screenings using the hospital screening instrument (*κ* = 0.25–0.54; Table 1). Hospital staff identified a lower proportion of patients with needs related to medication affordability, housing instability, food insecurity, transportation needs, and access to mental health services and a higher proportion with needs related to usual source of medical care. Results were similar when data were stratified by study year and staff role completing HRSN screening (Appendix Tables 2, 3a, and 3b). Conversely, research staff demonstrated substantial agreement in screenings for housing instability, food insecurity, and transportation needs using the two different instruments (*κ* = 0.64–0.81; Table 2).

**Table 1:** Comparison of health‐related social needs identified by hospital staff and research staff.

Health‐related social need domain, *n* (%)^a^	Hospital staff (*n* = 413)	Research staff (*n* = 413)	*κ*
Usual source of care^b^	57 (13.8)	32 (7.8)	0.54
Medication affordability	34 (8.2)	48 (11.6)	0.33
Housing	28 (6.8)	52 (12.6)	0.40
Food	37 (9.0)	68 (16.5)	0.45
Transportation	34 (8.2)	65 (15.7)	0.40
Mental health	38 (9.2)	69 (16.7)	0.25

^a^ Number and percent represent those with a need in that domain.

^b^ Doctor or clinic where patient usually goes for medical care.

**Table 2:** Comparison of health‐related social needs identified by research staff using two different instruments.

Health‐related social need domain, *n* (%)^a^	Hospital instrument (*n* = 413)	AHC HRSN (*n* = 413)	*κ*
Housing	52 (12.6)	37 (9.0)	0.64
Food	68 (16.5)	72 (17.4)	0.81
Transportation	65 (15.7)	77 (18.6)	0.80

Abbreviation: AHC HRSN, Accountable Health Communities Health‐Related Social Needs questionnaire, an instrument developed by the Centers for Medicare and Medicaid Services.

^a^ Number and percent represent those with a need in that domain. Hospital instrument developed by improvement team and based on review of published instruments.

## DISCUSSION

Our study revealed fair to moderate agreement between hospital staff and research staff HRSN screenings using the same instrument, yet substantial agreement for screenings conducted by research staff using two different instruments. Hospital staff generally identified a smaller proportion of patients with needs than research staff. Our findings are important because policies now require hospitals to assess patients' HRSNs and provide information about community resources and support services. Yet policymakers provide minimal guidance on how screening should be conducted and allow hospitals to use self‐selected screening instruments administered by staff roles and at points of time of their choosing. Our findings show that the manner in which HRSN screening is conducted influences its effectiveness.

There are several potential reasons that agreement was fair to moderate between hospital staff and research staff HRSN screenings using the same instrument, and that hospital staff identified a smaller proportion of patients with needs. Nurses performed HRSN screening during patients' admission assessments. Nurses may have prioritized the assessment and management of acute symptoms, administration of urgent treatments, and coordination of care with other healthcare professionals. Prior research has found that, while healthcare professionals generally support efforts to identify and address HRSNs, they express concerns about workflow disruptions, added workload, and changing role responsibilities.^7^, ^8^ Nurses in our study may have perceived HRSN screening as adding to an already high workload or poorly fitting into their workflow. Patients may have prioritized rapid treatment of symptoms and medical conditions over HRSN screening. Patients may have also been reluctant to share social needs due to fear of bias by clinical team members. Prior research has shown that some patients are concerned that HRSN data collection may result in discrimination and stigma and influence the quality of care received.^9^ In our study, research staff conducted HRSN screening on patients' second or third hospital day, a point at which patients may have been more likely to be clinically stable and receptive to screening.

Our findings have implications for hospitals seeking to optimize screening to identify and address social needs. In determining an optimal approach, hospitals need to consider the tradeoffs between efficiency and effectiveness. Having nurses conduct screening during admission assessments may ensure that all patients are screened and allow time to address identified needs, but may underestimate the true proportion with social needs.

Our findings have also implications for potential strategies to modify risk adjustment for patient outcomes.^10^ Research has shown that hospitals serving socially disadvantaged patients may be penalized using current strategies for risk adjustment in Medicare's Hospital Readmission Reduction Program.^11^, ^12^ One approach to improving social risk adjustment is to use hospital HRSN screening data (e.g., using Z‐codes).^13^ Our study suggests that, without improved screening methods, this approach would also fail to optimally control for social risk.

Our study has several limitations. First, the study was conducted on general medical services in a single hospital. Second, although based on published instruments, the hospital screening instrument did not undergo formal psychometric testing. Finally, our analysis is limited in that it only compared patients who agreed to participate in the study. We excluded patients with cognitive impairment, limited English proficiency, and other communication barriers; populations at risk for social needs.

In conclusion, this study suggests that the effectiveness of HRSN screening is influenced by how the screening is conducted. Screening conducted by hospital staff may underestimate the proportion of patients with social needs. Further research is needed to characterize barriers to effective HRSN screening, which may include workload, workflow, and patients' reluctance to share HRSN information with hospital staff.

## CONFLICT OF INTEREST STATEMENT

The authors declare no conflicts of interest.

## Supporting information

Hospitalized Patients HRSN‐A Comparison of Screening‐APPENDIX‐5‐25‐2025‐clean.

Hospitalized Patients HRSN‐A Comparison of Screening STROBE checklist cross‐sectional.
