# Supplementary material for: Hospitalized patients' health‐related social needs: A comparison of screenings conducted by hospital staff and research staff
Source: J Hosp Med. 2025 Aug 20;21(2):171–4. doi: 10.1002/jhm.70164 (PMC12865252; doi:10.1002/jhm.70164)
Supplement: Supplementary file 1 — Hospitalized Patients HRSN‐A Comparison of Screening‐APPENDIX‐5‐25‐2025‐clean. [file JHM-21-171-s001.docx]

**APPENDIX**

**Structured Interview to Identify Hospitalized Patients’ Health-Related Social Needs**

Introductory Text (for interviews conducted by research staff)

You are receiving treatments in the hospital to help improve your health. We know that many things can affect your health. Often, factors at home and in the community can affect one's health. We are doing a research study to learn more about patients' health-related social needs. I would like to ask you a series of questions related to social needs. This interview should take 10 to 15 minutes. If you feel uncomfortable, you may skip a question.

Health-Related Social Needs Screening Items Developed by Hospital Quality Improvement Team

1. Do you have a doctor or clinic where you usually go for medical care? (yes/no, no reflects usual source of care need)
2. Do you have trouble getting medications, medical supplies, or paying for medication co-pays on a regular basis? (yes/no, yes reflects medication affordability need)
3. Are you concerned about having a safe and reliable place to live? (yes/no, yes reflects housing instability)
4. Have there been times when your food ran out and you didn’t have enough money to get more? (yes/no, yes to either question 4 or 5 reflects food insecurity need)
5. Have there been any times recently that you worried whether your food would run out before you got money to buy more? (yes/no, yes to either question 4 or 5 reflects food insecurity need)
6. Do you have trouble getting transportation to medical appointments? (yes/no, yes reflects transportation need)
7. Would you like help finding professional services to help with stress, depression, anxiety or other mental health concerns? (yes/no, yes reflects need for mental health services)

Health-Related Social Needs Screening Items from the Accountable Health Communities Health-Related Social Needs questionnaire

Housing Instability

1. What is your living situation today? (response to either of the latter options indicates presence of housing instability)

- I have a steady place to live
- I have a place to live today, but I am worried about losing it in the future
- I do not have a steady place to live (I am temporarily staying with others, in a hotel, in a shelter, living outside on the street, on a beach, in a car, abandoned building, bus or train station, or in a park)

Food Insecurity: Some people have made the following statements about their food situation. Please answer whether the statements were OFTEN, SOMETIMES, or NEVER true for you and your household in the last 12 months. (response of often true or sometimes true to either question 2 or 3 indicates presence of food insecurity)

1. Within the past 12 months, you worried that your food would run out before you got money to

buy more.

- Often true
- Sometimes true
- Never true

1. Within the past 12 months, the food you bought just didn't last and you didn't have money

to get more.

- Often true
- Sometimes true
- Never true

Transportation Need

1. In the past 12 months, has lack of reliable transportation kept you from medical appointments, meetings, work or from getting things needed for daily living? (response of yes indicates presence of transportation need)

- Yes
- No

**Figure.** Consolidated Standards of Reporting Trials (CONSORT) flow diagram of patients eligible, recruited, and included in analysis.

2,432 patients excluded

- 1,296 due to cognitive impairment*
- 953 due to Limited English Proficiency
- 183 due to communication barrier

838 patients declined to participate

1,055 patients completed HRSN screens conducted by research staff

427 patients completed HRSN screens conducted by research staff and hospital staff

14 patients excluded due to missing data elements

413 patients included in the analysis

4,325 patients on general medical units at study hospital assessed for eligibility

*Cognitive impairment includes dementia, delirium, psychosis, or mania.

628 patients excluded due to missing HRSN screens conducted by hospital staff

**Table 1.** Patient Demographic Characteristics

| Characteristic, n (%) or mean (SD) | | N=413 |
| --- | --- | --- |
| Age | | 60.1 (16.7) |
| Sex | |  |
|  | Female | 197 (47.7) |
|  | Male | 216 (52.3) |
| Race | |  |
|  | White | 250 (60.5) |
|  | Black | 138 (33.4) |
|  | Asian | 6 (1.5) |
|  | Other | 8 (1.9) |
|  | Unknown/prefer not to say | 11 (2.7) |
| Ethnicity | |  |
|  | Hispanic or Latino | 38 (9.2) |
|  | Not Hispanic or Latino | 360 (87.2) |
|  | Unknown/prefer not to say | 15 (3.6) |
| Highest level of education completed | |  |
|  | Did not complete high school | 25 (6.1) |
|  | High school | 192 (46.5) |
|  | 4-year college | 112 (27.1) |
|  | Advanced degree | 77 (18.6) |
|  | Unknown/prefer not to say | 7 (1.7) |
| Payer | |  |
|  | Medicare | 223 (54.0) |
|  | Private | 118 (28.6) |
|  | Medicaid | 69 (16.7) |
|  | Other/self-pay | 3 (0.7) |

**Table 2.** Health-Related Social Needs Agreement between Hospital Staff and Research Staff by Year of Study

| Health-Related Social Need Domain, n (%) * | Year 1  N=156 | Year 2  N=70 | Year 3  N=187 |
| --- | --- | --- | --- |
| Usual source of care | 0.63 | 0.46 | 0.49 |
| Medication affordability | 0.34 | 0.41 | 0.28 |
| Housing | 0.48 | 0.37 | 0.35 |
| Food | 0.38 | 0.43 | 0.50 |
| Transportation | 0.50 | 0.30 | 0.37 |
| Mental health | 0.42 | 0.25 | 0.10 |

*Agreement assessed using Kappa statistic

**Table 3a.** Comparison of Health-Related Social Needs Identified by Nurses and Research Staff

| Health-Related Social Need Domain, n (%) * | Nurses (n=354) | Research Staff (n=354) | Kappa |
| --- | --- | --- | --- |
| Usual source of care | 51 (14.4) | 28 (7.9) | 0.56 |
| Medication affordability | 26 (7.3) | 40 (11.3) | 0.33 |
| Housing | 23 (6.5) | 43 (12.2) | 0.34 |
| Food | 32 (9.0) | 58 (16.4) | 0.47 |
| Transportation | 26 (7.3) | 54 (15.3) | 0.36 |
| Mental health | 33 (9.3) | 59 (16.7) | 0.23 |

* Number and percent represent those with a need in that domain.

**Table 3b.** Comparison of Health-Related Social Needs Identified by Social Workers and Research Staff

| Health-Related Social Need Domain, n (%) * | Social Workers (n=59) | Research Staff (n=59) | Kappa |
| --- | --- | --- | --- |
| Usual source of care | 6 (10.2) | 4 (6.8) | 0.35 |
| Medication affordability | 8 (13.6) | 8 (13.6) | 0.28 |
| Housing | 5 (8.5) | 9 (15.3) | 0.68 |
| Food | 5 (8.5) | 10 (17.0) | 0.32 |
| Transportation | 8 (13.6) | 11 (18.6) | 0.56 |
| Mental health | 5 (8.5) | 10 (17.0) | 0.32 |

* Number and percent represent those with a need in that domain.
